# Decomposition pattern and insect colonization in two cases of suicide by hanging

**DOI:** 10.1080/20961790.2017.1418622

**Published:** 2018-02-13

**Authors:** Valentina Bugelli, Mirella Gherardi, Martina Focardi, Vilma Pinchi, Stefano Vanin, Carlo Pietro Campobasso

**Affiliations:** a Department of Medicine and Health Sciences (DiMeS), University of Molise, Campobasso, Italy; b Department of Medicine and Health Sciences, Forensic Science Section, University of Florence, Firenze, Italy; c Prevention Department, SC Medicina Legale, AUSL Valle D'Aosta, Aosta, Italy; d Department of Biological Sciences, School of Applied Sciences, University of Huddersfield, Huddersfield,UK; e Department of Experimental Medicine, University of Campania “L. Vanvitelli”, Naples, Italy

**Keywords:** Forensic science, forensic entomology, forensic taphonomy, decomposition, hanging, postmortem interval

## Abstract

Hanging is one of the most common methods of suicide worldwide. Despite the high incidence, only a little knowledge about the pattern of cadaver colonization by insects on hanging corpse is available. Different types of hanging can alter the body decomposition process as well as the pattern and rate of insect colonization. Two case studies where the hanging occurred with a similar postmortem interval of 34 days are described. The two bodies showed different patterns of insect colonization and decomposition scored using the Total Body Score (TBS) and the TBS for hanging (TBS_hang_). The first case was about the body of a 24-years-old male, with mummification of the unclothed upper anatomical parts. A TBS of 14 and a TBS_hang_ of 18 were assigned. The second body, belonging to a 15-years-old male, was found pre-skeletonized lying on the ground with the skull disarticulated. A TBS of 31 and a TBS_hang_ of 32 were assigned. Average temperatures of (21.5 ± 2.5) °C for the first body and (25.1 ± 2.7) °C for the second body were recorded in the 34 days preceding the bodies’ discovery for a total of 731 and 853 Accumulated Degree Days (ADD) respectively. According to previous studies, the different decomposition patterns were related to temperatures of exposition and to the diversity of arthropod community found on the bodies because of the different types of hanging: totally suspended *vs*. in contact with the soil. The limited insect activity caused by hanging explains the delay in decomposition of hanging bodies in which mummification can take place, especially on the upper body parts. In vertical body position, the body fluids accumulated in the lower body parts accelerating the desiccation of soft tissues on the upper parts. The effect of gravity can also explain the decrease of internal maggot mass as larvae easily fall from the hanging body to the drip zones below where they are unable to recolonize the body if totally suspended. Furthermore, in a hanging body a greater surface is exposed to wind and sun with a quicker skin drying preventing the additional Diptera colonization. This paper shows the weaknesses of scoring scales and regression models developed to predict ADD when irregular decomposition and mummification have not taken into account.

## Introduction

Hanging is one of the most common methods of suicide worldwide with an estimated fatality rate of over 70% [1]. The cause of death in hanging is mainly due to asphyxia secondary to compression of the neck structures. Hanging has been defined as a form of strangulation in which the pressure on the neck is applied by a constricting band tightened by the gravitational weight of the body or of body part [2]. Hanging is classified as complete or incomplete as the suspension can be often not high enough to keep the victim's feet or legs clear of the floor.

The most common place chosen by the suicidal victim is home, followed by isolated outdoor places where bodies may remain undiscovered for long time [1] allowing the development of advanced decomposition patterns. Despite the high incidence of suicide by hanging, only a little knowledge on the patterns of human cadaver colonization by insects on suspended corpses is available [3,4].

Based on the comparison of the decomposition patterns in hanging pigs and pig carcasses on the ground, it has been demonstrated that the suspended position can affect the rate of biomass removal and of decomposition as well as the overall insect activity [3,4]. In this regard, a new scoring scale for hanging pigs has been proposed [3] in order to include mummification in the evaluation of decomposition, a morphological feature not fully considered in the Total Body Score (TBS) by Megyesi et al. [5] on humans. Mummification and the suspended position of the carcass/body were considered the two main factors affecting both the delay in decomposition of hanging bodies secondary to the lower variety and the amount of insects compared with carcasses on the ground [3]. Several flaws with Megyesi et al. [5] have been recently emphasized mainly concerned with the statistical analysis and the temperature scale adopted in the original publication [6]. In this regard, a more appropriate regression model to predict accumulated degree days (ADD) from TBS have been proposed for casework and experimental studies [6].

In order to show the differences related to the necrophagous insect community and human decay pattern, two case studies are described. The two cases share the same manner of death (suicide by hanging) and the same postmortem interval (PMI) of 34 days. Despite these similarities, the bodies showed two completely different patterns of decomposition and insect colonization according to different temperatures of exposure (daily average temperature ranging between 21.5 °C and 25.1 °C).

A point-based system for the main three anatomical regions (head and neck, torso, limbs) was applied for scoring decomposition according to previous studies for bodies in contact with the ground and for bodies totally suspended [4,5]. The TBS scale proposed by Megyesi et al. [5] for bodies in contact with the ground has been used in comparison with the new one proposed specifically for hanging bodies (TBS_hang_) by Lynch-Aird et al. [3].

Case 1

**Figure 1.**
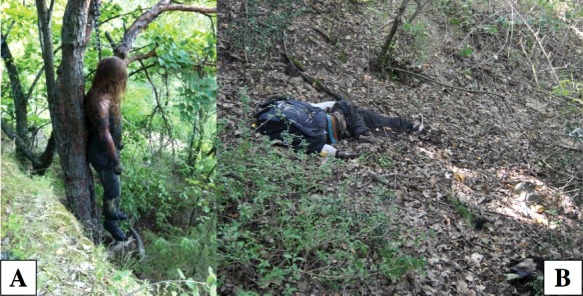
The remains of the two hanging bodies at the death scene: the body totally suspended in case 1 (A), the remains laying on the ground in case 2 (B).

**Figure 2.**
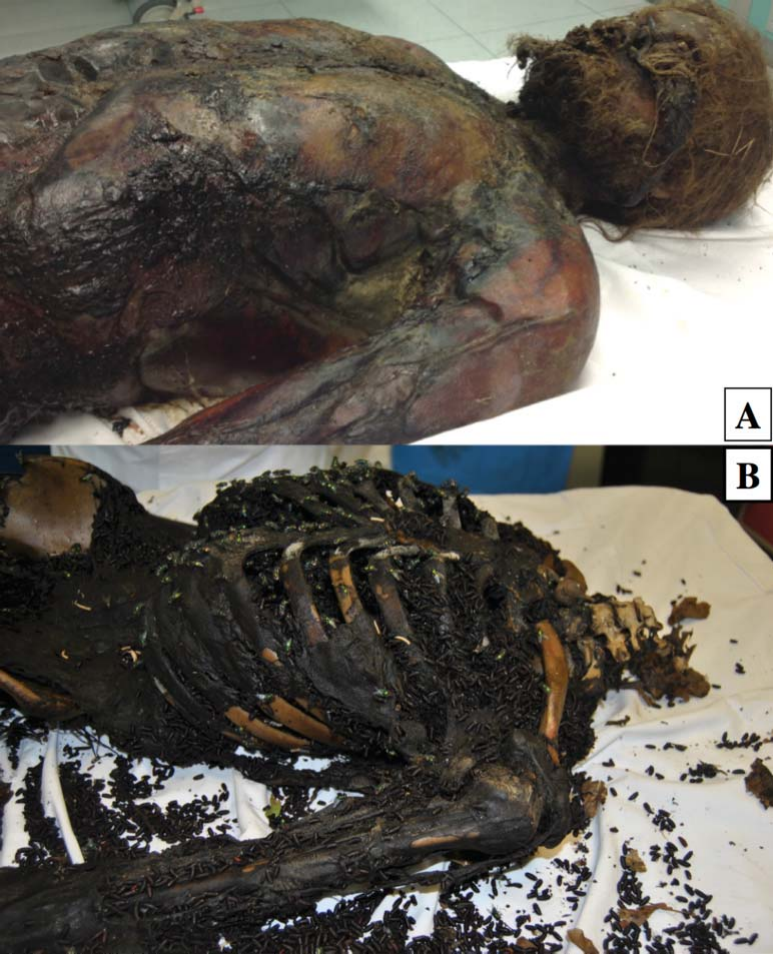
The decomposition pattern of the two hanging bodies: the mummified soft tissues of the upper part in case 1 (A), pre-skeletonization of the thorax and the neck in case 2 (B).

The body of a 24-years-old male was found in a woodland, in North Western Italy, in the middle of July, 34 days after his disappearance. The body was totally suspended from a branch of a big tree by a leather belt (Figure 1(A)). The feet were at about 1 m above the ground. At the drip zone, the ground directly beneath the hanging body, Diptera maggots falling from the corpse were found along with few Diptera pupae and puparia. At the external examination the body was preserved, except for the structures of the face, strongly affected by the feeding activity of the early larval stages. The mummification process took place in the remaining bare regions of the upper body part (scalp, thorax, upper arms). The mummification was clearly displayed by the desiccation and brittleness of the skin, which was stretched tightly across anatomic prominences (Figure 2(A)). Brown to black discolouration of flesh at the neck, with the skin having a leathery appearance and adherent to bones was found. Discolouration changing from green to black was observed at the bare trunk, post-bloating following the release of the abdominal gases. Marbling at the lower arms and lower limbs, hanging in usual position, with skin slippage on hands resulting in “glove formation” were also visible along with some epidermal maceration of the feet. Based on the above features, a TBS of 14 and a TBS_hang_ of 18 were assigned.

No lesions by blunt trauma or fractures of long bones were found at the X-ray examination. Hyoid bone was also intact, but thyroid and cricoid cartilages were fractured due to suspension. Vital signs of haemorrhages were observed on mummified skeletal muscles close to the bone fractures. Therefore, the cause of death was finally determined as suicidal hanging.

**Figure 3.**
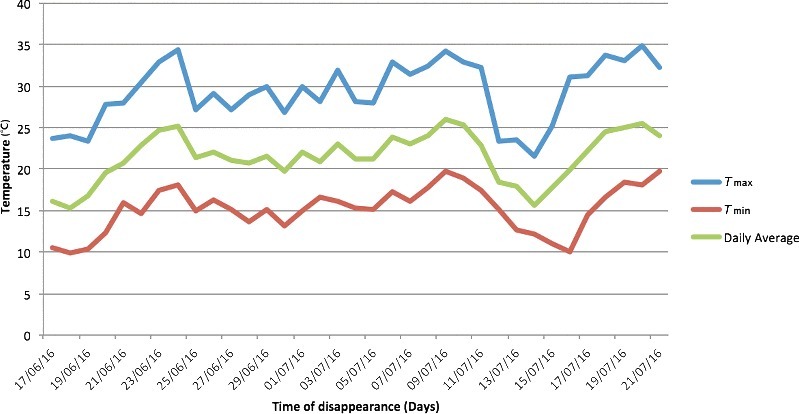
Temperatures recorded by the weather station (1.6 km far from the death scene) for the 34 days before the discovery of the body (*T*_max_: 34.9 °C; *T*_min_: 9.9 °C) in Case 1.

An overall mean temperature of (21.5 ± 2.5) °C was recorded by the weather station of Villenueve Saint Nicolas, 1.6 km far from the death scene, for the 34 days before the discovery of the body corresponding to 731 ADD. Temperature records are summarized in Figure 3. Temperature ranged from a maximum of 34.9 °C to a minimum of 9.9 °C.

Insect activity was visible only on the face where few Diptera larvae (1.5–1.8 mm in length) were recovered. A large amount of adults and active larvae of Coleoptera were also detected from the scalp. Beetles adults and larvae represented the predominant part of the insect community. Just few Diptera pupae and puparia were recovered in the drip zone below. The insect community recovered on the body and at the drip zone was identified according to species-specific morphological identification keys as summarized in Table 1. The specimens of Diptera useful for the estimation of the minimum postmortem interval (PMI_min_) were empty puparia of *Phormia regina* (*Meigen*) and *Lucilia sericata* (*Meigen*) (Diptera: Calliphoridae). Based on relevant developmental data published for these species [7,8], *L. sericata* needs from a minimum of 14.1 days (at 22 °C) to a maximum of 18.7 days (at 20 °C) to complete the development (eggs-adults) and *P. regina* from a minimum of 15.2 days (at 22 °C) to 18.8 days (at 23 °C) to reach the adult stage [9]. Although it is widely accepted that there may be significant differences between temperatures experienced at the death scene and those collected retrospectively from weather stations [10,11], unfortunately no data loggers were placed on site and, therefore, no regression relationship was derived between ambient temperatures at site and weather station to make a retrospective correction. Based on fly species, and a mean temperature of 21.5 °C, a PMI_min_ of 14–18 days was estimated. But the development times of fly species could be much longer than that as caution should be exercised when applying development data from studies performed at consistent temperatures to outdoor scenes where the temperature has fluctuated [11]. In fact, in this case, active larval stage of *Dermestes frischii* (*Krugelann*) indicated a longer PMI_min_ of no less than 30 days. Based on developmental data [12], *D. frischii* needs at least 29.7 days to get pre-pupa larval stage at 23 °C as a complete life cycle of this Coleoptera has been observed in 111–115 days at 20 °C [13].

Case 2

**Table 1.** Insects collected on bodies at the death scene and during the autopsy.

Taxon	Case 1	Case 2
*Lucilia sericata* (Meigen, 1826) (Diptera: Calliphoridae)	Larvae 3rd instar, puparia, adults	–
*Phormia regina* (Meigen, 1826) (Diptera: Calliphoridae)	Larvae 3rd instar, puparia	–
*Chrysomya albiceps* (Weidemann, 1819) (Diptera: Calliphoridae)	–	Pupae, puparia
*Nasonia vitripennis* (Walker,1836) (Hymenoptera: Pteromalidae)	–	Adults
*Hermetia illucens* (Linnaeus, 1758)(Diptera: Stratiomyidae)	–	Pupae
*Creophilus maxillosus* (Linnaeus, 1758)(Coleoptera: Staphylinidae)	Adults	–
*Dermestes frishii* (Kugelann, 1792)(Coleoptera: Dermestidae)	Pre-pupa, exuviae, adults, larvae	–

-: not found.

The body of a 15-years-old male was found in a forest, in the Centre of Italy, in a suburban area of Florence (Tuscany) at the beginning of October. The man was last seen alive at the end of August, 34 days before his body discovery. The body, fully clothed, was found lying on his left side in a wooded shady area (Figure 1(B)). A noose with short, curling black hairs still attached, suggesting the body may have fallen to the ground during decomposition, was present on the tree above the body. Clothes were covered by pupae and empty puparia of Diptera. The head was disarticulated and partially skeletonized and was found approximately 2 m far from the body. The part of the head lying on the floor was completely skeletonized, while the other side showed some dried soft tissues still attached. The body was almost completely skeletonized (Figure 2(B)): dried soft tissues were also present at the extremities of arms and legs. Radiographs disclosed the fracture of the right greater horn of the hyoid bone and no other bone fractures or evidence of blunt trauma or signs of gunshot injuries or knife wounds. A TBS of 31 and a TBS_hang_ of 32 were assigned. In the absence of evidence of trauma and other pathological findings, the cause of death was finally determined as suicidal hanging according to the scene findings and the hyoid bone fracture.

**Figure 4.**
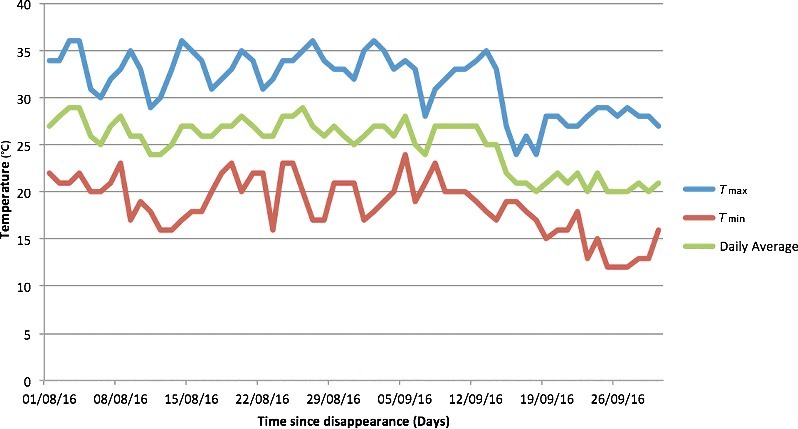
Temperatures recorded by the weather station (5.1 km far from the death scene) for the 34 days before the discovery of the body (*T*_max_: 30.0 °C; *T*_min_: 12.0 °C) in Case 2.

An overall mean temperature of (25.1 ± 2.7) °C (max. 30.0 °C, min. 12.0 °C) was recorded by the weather station of the Airport of Florence, 5.1 km far from the death scene, during the 34 days before the discovery of the body corresponding to 853 ADD. Temperature records are summarized in Figure 4. The skeletonized chest was largely colonized by Diptera larvae 1.8 mm in length. The insect community recovered at the drip zone and on the body was identified as specimens at different developmental stages belonging to Diptera and Hymenoptera as summarized in Table 1. Empty puparia of *Chrysomya albiceps* (*Weidemann*) were considered for the PMI_min_ estimation. Several puparia of *C. albiceps* showed the characteristic circular emergence holes typical of a *Nasonia vitripennis* (*Walker*) parasitization. Unfortunately, also in this case, no ambient temperature correction was possible as no data loggers were placed on site. Based on the published developmental data, larvae of *C. albiceps* need at 25 °C at least 8.1 days to reach the pupal stage and additional 13 days for the eclosion [14]. *Nasoniavitripennis* emergence holes are evidence of an additional time interval needed for the parasitic wasp to complete the development ranging from a minimum of 14.8 days (at 25 °C) to 22.5 days (at 20 °C) [15]. Based on the above data, a PMI_min_ of 15–22 days was estimated but the development times of fly species could be much longer than that according to the inappropriate utilization of fluctuating temperatures from weather station, without any retrospective correction, to studies providing development data at constant temperatures. In fact, pupae of *Hermetia illucens* (*Linnaes*) indicated a longer PMI_min_ of about 30 days. Based on developmental data, *H. illucens* larvae need at least 31 days at 27.8 °C to complete the development [16–18].

## Discussion

Decomposition is a sequential process influenced by a complex interaction of biotic factors (i.e. bacteria and insects) and abiotic factors (i.e. weather conditions) among which temperature and insect activity are the most influential [19]. Recently published studies have defined the ecological mechanisms of decomposition emphasizing the strong interactions between the microbial community, the products of decay and the necrophagous insects [20–22]. The postmortem changes can be still divided into two main groups: destructive (i.e. putrefaction) and conservative phenomena, depending mainly on the environmental conditions (i.e. maceration in immersed bodies, mummification in hot dry environment) [23]. Therefore, immediate postmortem changes are for the most part the result of a competition between putrefaction and desiccation [24]. In this regard, the two case studies are representative of such postmortem destructive or conservative processes.

The different decay patterns are clearly indicated in the two hanging case studies by the different assigned TBSs: the first body showed irregular decomposition with mummification of the upper anatomical bare parts and early postmortem changes of the lower torso and limbs while the second case, fully clothed, was pre-skeletonized with disarticulation of the skull (Figure 2). Based on the above features, TBSs of 14 and 31 were assigned, respectively, corresponding to a TBS_hang_ of 18 and 32, although the similarities shared by the bodies concerning the same manner of death (suicidal hanging) and the same PMI (34 days). One of the predominant reasons explaining the different decomposition patterns and insect community associated with human remains is represented by the different weather conditions of exposition with a daily average temperature of 21.5 °C and 25.1 °C, respectively equating 731 and 853 ADD. Since ADD incorporate time and heat in a single value, they are the preferred variables rather than actual days for calculations quantifying the PMI especially for research purposes, not only in forensic entomology but also in forensic anthropology [25]. ADD should be used with caution as temperature data are not always created equal, reflecting exactly temperature conditions at the discovery site [11]. In both case studies, unfortunately no ambient temperature corrections were possible as no data loggers were placed on site.

However, in the present case studies, it is worth of mentioning that a gap of few grades centigrade caused big differences in ADD values affecting deeply the decomposition pattern and insect colonization. The accelerated decomposition pattern observed in the second case was certainly promoted by the daily average temperature (25.1 °C) being higher than the first case (21.5 °C), especially in the first couple of weeks. The high temperatures recorded in the second case (equating 853 ADD) probably accelerated the development rate of insect and, therefore, the decomposition rate of the cadaver. In fact, according to Simmons et al. [26], the insect presence can be considered the primary agent affecting the pattern of decomposition on surface remains. However, it also true that the reverse relationship between putrefaction and desiccation can affect insect colonization and succession as observed in the two case studies presented.

Previous studies on arthropod succession on animal and human remains have demonstrated a strong relationship between different insect communities and specific decomposition patterns [27–29]. Arthropods are able to detect volatile organic compounds (VOCs) produced by the breakdown of soft tissues during the decomposition and use them to locate the more suitable food resource for their offspring [30,31]. VOCs profiles differ over the course of human decay affecting the successional pattern of insect related to the human remains. Therefore, it can be assumed that insect colonization was similar in the case studies and started in their natural openings (i.e. mouth, nose, eyes) or in close proximity of the hanging mark. Later, the decomposition process changed in the first case toward the mummification and in the second case toward putrefaction affecting insect colonization and decomposition pattern.

Temperature and type of environment (woodland in the first case, a forest in the second one) as well as access of insects are well known as the most important variables influencing putrefaction or preservation of soft tissues such as mummification [19,32]. But temperatures alone cannot explain the significant differences between the two bodies. Additional reasons have to be considered, among which mainly the body position at the death scene (Figure 1). In the first case, the suspension was complete while in the second case, the human remains probably fell down during decomposition, once the neck was completely skeletonized by insect activity. Therefore, the contact with the ground as well as the hanging position of the body can be crucial for the decomposition pattern and insect colonization as already emphasized in previous studies [3,4].

Goff and Lord [33] already found that hanging could alter the insect colonization pattern excluding soil-dwelling taxa, thus changing the drying pattern of the body. An experimental study on animal carcasses [4] revealed also a delayed progression through the physical stages of decomposition and a slower biomass removal rate in hanging pigs compared with pig carcasses in contact with the ground. The prolonged time for each stage of decomposition was mainly related to the inability of larvae that fell to the ground or the ground-dwelling insects to regain access to the carcass that prevented the formation of a well-established internal maggot mass [4]. In a larger experimental study based on 20 pig carcasses [3], the delay in the decomposition in hanging animals was confirmed and related to a lower variety and quantity of insects compared with pigs on the surface of the ground. However, in the first case in the present paper, the insect community on the decomposing body was mainly represented by adult and active larval stage of Dermestidae. In fact, larder beetles’ activity is commonly associated with the advanced stage of decay contributing to the drying out of liquefied tissues.

Lynch-Aird et al. [3] demonstrated that the decomposition pattern of hanging bodies is markedly different due to the mummification process, a feature not fully considered in the Megyesi et al.'s scale [5]. Mummification is a process of preservation quite common, especially in forensic cases of hanging like those described earlier. Mummified skeletal muscles have occurred in several high-profile cases providing useful details for the death investigation such as vital signs of haemorrhages [34] like those observed at the neck of the first body. In hot, dry climates, the entire body or discrete portions can dehydrate rapidly and may go into mummification rather than decomposition [35]. This process includes mainly the drying of the soft tissues instead of liquefying putrefaction.

In the first case, the mummification process has contributed significantly to change the value of TBS from 14 to a TBS_hang_ of 18 according to Lynch-Aird et al. [3]. Unfortunately, using the old and new regression equations proposed to predict ADD [5,6], the prediction intervals for ADD from both TBS and TBS_hang_ values do not fit the daily average temperatures collected by the nearest weather stations in both cases. Several factors can explain the distance in predicting ADD from quantitative scales among which worth of mentioning are the subjectivity of examiners when using scoring systems and the lack of accuracy of retrospective temperatures recorded by weather stations with real temperatures of exposition on-site [10,36]. It is well known that blind utilization of temperature data from weather stations can be inaccurate as there may be significant differences between temperatures recorded at the death scene and those collected retrospectively from stations [11]. Such differences and the missing retrospective corrections may explain in the present case studies the inaccuracy of the assigned TBS values in predicting ADD. However, currently it is controversial the benefit of using retrospective corrections for short and long-term correlations between site-specific and weather station temperature data [25,37]. It has been also observed that scoring scales and regression equations derived for predicting ADD seem to be of little help in forensic practice [38,39] because of the so many factors affecting human decay and insect colonization among which irregular decomposition and mummification need to be taken into account [35].

In mummification, the skin has a brown to black leathery appearance, as observed in the first body totally suspended, while the internal organs continue to deteriorate, often reduced to a blackish-brown putty-like consistency. A natural mummification process usually needs 6–12 weeks but sometimes under ideal environmental conditions a body external surface can mummify in as little as 2–3 weeks [35,40]. The onset of mummification in indoor cadavers can be even faster than those found outdoors as observed in a retrospective study in Arizona [40,41]. A head-down position like the hang position can contribute to determine a rapid dehydration of the outer and upper surfaces of human remains and, therefore, the delay in decomposition of hanging bodies due to the onset of mummification. In hanging bodies, partially or totally suspended, as well as in bodies in a sitting position [42], the gravitational effect related to vertical body position causes the body fluids falling into the lower anatomical parts. Therefore, the fluid deprivation in the upper anatomical parts can accelerate the desiccation of soft tissues and the onset of mummification. Furthermore, in bodies totally suspended by hanging, the greater surface area exposure to wind and sun (such as the bare shoulder of the first body) can dry out quickly the skin and underlying soft tissues preventing further insect colonization.

Larval infestation for most of the blowflies is usually hindered by the rapid dehydration of the skin surface, facilitated in hanging bodies by the early putrefactive fluids falling on the drip zone. The effect of gravity can also explain the decrease of internal maggot mass as larvae easily fall from the hanging body to the drip zone below where they are unable to get back to the body if it is totally suspended like was observed in the first case. The absence of a well-established maggot mass can be also explained due to the predatory activity of birds, ants and beetles. Sometimes the presence of ants [43] and larder beetles [44] can be considered a significant factor affecting the decomposition pattern and insect colonization. Therefore, in the first case, several factors (i.e. the effect of gravity, the quick dehydration of unclothed upper tissues, the absence of a well-established maggot mass) contributed to maintain the body in the hanging position, representing a preservative decay process while the second case is quite representative of a destructive decay process. In this latter case, the body, fully clothed, was found pre-skeletonized on the surface of the ground with the skull disarticulated after 34 days from disappearance.

Human decomposition is a quite complex interaction between intrinsic and extrinsic factors [23] producing individual pattern of decomposition and insect colonization. According to Parks [45], a unique decomposition model cannot fit all potential cases of forensic interest due to irregular decomposition taking place in response to environmental conditions, particularly temperature, humidity, ventilation, etc. Anomalies of decomposition can easily occur because of ante-mortem injuries or postmortem scavenging of human remains due to insects or larger terrestrial and aquatic animal life such as wolfs, dogs, birds, fishes, crustaceans, mollusks, etc. [35].

In this regard, it can be assumed that in the second case, after oviposition in the natural openings or close to the hanging mark, Diptera larvae actively feeding on tissues started the break-down of the body and consumption of soft tissues, especially at neck level, and most likely caused the fall of the body on the drip zone. Therefore, it is possible that in the second fully clothed body, the collar gathered enough maggots to cause an accelerated consumption of the tissue of the neck, and the cadaver to finally fall down to the ground before mummification took place. Once on the ground surface, the decomposition rate would be faster because maggots that had dropped to the ground easier were able to regain access to the cadaver with no restricted access to beetles. On the ground, the subsequent waves of insects could sustain a fast decomposition rate based on actively feeding insect community.

## Conclusion

The two case studies presented in this paper shared the same manner of death (suicide by hanging) and the same PMI (34 days), but there were significant differences in the pattern of decomposition and insect colonization. The season (beginning of summer in the first case, end of summer/beginning of autumn in the second case), the geographical location of death (North West Italy in the first case, Tuscany in the second one) and the type of habitat (woodland in the first case, a forest in the second one) contribute to these differences. However, the main differences were the temperatures of exposure (respectively equating 731 and 853 ADD) and the position in which the bodies were found on the death scene (the first one totally suspended, the other on the lying on the ground).

A new scoring scale for hanging bodies has been proposed by Lynch-Aird et al. [3] in order to include more features related to mummification not fully provided by Megyesi's system [5]. And a more appropriate regression model to predict ADD from TBS of terrestrially deposited bodies has been also developed for casework and experimental studies [6]. Scoring scales have contributed significantly to provide a more objective picture of human decomposition, promoting the inclusion of statistics in a taphonomic discipline primarily descriptive [3,5,6,46]. However, in spite of the efforts expended in establishing accurate PMI estimates by using scoring scales, the regression equations to predict ADD (mostly based on animal models) seem unable to offer reliable results in forensic practice dealing with human corpses. In addition, it seems, from the cases evaluated in this paper, that scoring scales are of little help in forensic practice because of the many factors affecting human decay and insect colonization [38,39]. Therefore, more validation of these methods is necessary before they can be applied to courtroom proceedings especially for the inclusion of statistics in human models of decay. Integrating all the taphonomic variables into a single decomposition model or a single mathematical equation seems actually a “mission impossible” in forensic entomology [38,47] and forensic pathology practice.

## References

[1] Gunnell D, Bennewith O, Hawton K, et al. The epidemiology and prevention of suicide by hanging: a systematic review. Int J Epidemiol. 2005;34:433–442. 15659471 10.1093/ije/dyh398

[2] Sauvageau E, Boghossian E Classification of asphyxia: the need for standardization. J Forensic Sci. 2010;55:12569–12567. DOI:10.1111/j.1556-4029.2010.01459.x 20561144

[3] Lynch-Aird J, Moffatt C, Simmons T Decomposition rate and pattern in hanging pigs. J Forensic Sci. 2015;60:1155–1163. 26249126 10.1111/1556-4029.12796

[4] Shalaby OA, De Carvalho LML, Goff ML Comparison of patterns of decomposition in a hanging carcass and a carcass in contact with soil in a xerophytic habitat on the island of Oahu. Hawaii J Forensic Sci. 2000;45:1267–1273. 11110180

[5] Megyesi MS, Nawrocki SP, Haskell NH Using accumulated degree-days to estimate the postmortem interval from decomposed human remains. J Forensic Sci. 2005;50:1–9. 15932096

[6] Moffatt C, Simmons T, Lynch-Aird J An improved equation for TBS and ADD: establishing a reliable postmortem interval framework for casework and experimental studies. J Forensic Sci. 2016;61:S201–S207. 26293169 10.1111/1556-4029.12931

[7] Grassberger M, Reiter C Effect of temperature on *Lucilia**sericata* (Diptera: Calliphoridae) development with special reference to the isomegalen-and isomorphen-diagram. Forensic Sci Int. 2001;120:32–36. 11457606 10.1016/s0379-0738(01)00413-3

[8] Byrd JH, Allen JC The development of the black blow fly, Phormia regina (Meigen). Forensic Sci Int. 2001;120:79–88. 11457615 10.1016/s0379-0738(01)00431-5

[9] Anderson GS Minimum and maximum development rates of some forensically important Calliphoridae (Diptera). J Forensic Sci. 2000;45:824–832. 10914578

[10] Archer MS The effect of time after body discovery on the accuracy of retrospective weather station ambient T°C corrections in forensic entomology. J Forensic Sci. 2004;49:553–559. 15171176

[11] Dabbs GR Caution! All data are not created equal: the hazards of using national weather service data for calculating accumulated degree days. Forensic Sci Int. 2010;202:e49–e52. DOI:10.1016/j.forsciint.2010.02.024 20303684

[12] Lambiase S, Murgia G, Sacchi R, et al. Effects of different temperatures on the development of *Dermestes**Frischii* and *Dermestes**undulatus* (Coleoptera, Dermestidae): comparison between species. J Forensic Sci. 2017 DOI:10.1111/1556-4029.13580 28631278

[13] Howe RH A summary of estimate of optimal and minimal condition for population increase of some stored products insects. J Stored Prod Res. 1965;1:177–184.

[14] Grassberger M, Friedrich E, Reiter C The blowfly *Chrysomya**albiceps* (Wiedemann) (Diptera: Calliphoridae) as a new forensic indicator in Central Europe. Int J Leg Med. 2003;117:75–81. 10.1007/s00414-002-0323-x 12690503

[15] Grassberger M, Frank C Temperature‐related development of the parasitoid wasp *Nasonia**vitripennis* as forensic indicator. Med Vet Entomol. 2003;17:257–262. 12941009 10.1046/j.1365-2915.2003.00439.x

[16] Tomberlin JK, Sheppard DC, Joyce JA Selected life-history traits of black soldier flies (Diptera: Stratiomyidae) reared on three artificial diets. J Entomol Sci. 2002;37:345–352.

[17] May BM The occurrence in New Zealand and the life-history of the soldier fly *Hermetia**illucens* (L.) (Diptera: Stratiomyidae). NZJ Sci. 1961;4:55–65.

[18] Lord WD, Goff ML, Adkins TR, et al. The black soldier fly *Hermetia**illucens* (Diptera: Stratiomyidae) as a potential measure of human postmortem interval: observations and case histories. J Forensic Sci. 1994;39:215–222. 8113702

[19] Mann R, Bass W, Meadows L Time since death and decomposition of the human body: variables and observations in case and experimental field studies. J Forensic Sci. 1990;35:103–111. DOI:10.1520/JFS12806J 2313251

[20] Pechal JL, Crippen TL, Benbow ME, et al. The potential use of bacterial community succession in forensics as described by high throughput metagenomic sequencing. Int J Leg Med. 2014;128:193–205. 10.1007/s00414-013-0872-1 23749255

[21] Tawni L, Crippen M, Benbow E, et al. Microbial interactions during the carrion decomposition. In: Benbow ME, Tomberlin JK, Tarone AM, editors. Carrion ecology, evolution, and their applications. Boca Raton (FL): CRC Press; 2016 p. 37–63.

[22] Tomberlin JK, Benbow ME, Barnes KM, et al. Arthropod-microbe interactions on vertebrate remains: potential applications in the forensic sciences. In: Carter DO, Tomberlin JK, Benbow ME, Metcalf JL, editors. Forensic microbiology. Hoboken (NJ): Wiley; 2017 p. 274–311.

[23] Campobasso CP, Di Vella G, Introna F Factors affecting decomposition and Diptera colonization. Forensic Sci Int. 2001;120:18–27. 11457604 10.1016/s0379-0738(01)00411-x

[24] Micozzi MS Postmortem change in human and animal remains: a systematic approach. Springfield (IL): CC Thomas Publishers; 1991.

[25] Dabbs GR How should forensic anthropologists correct national weather service temperature data for use in estimating the postmortem interval? J Forensic Sci. 2015;60:581–587. 25678225 10.1111/1556-4029.12724

[26] Simmons T, Cross PA, Adlam RE, et al. The influence of insects on decomposition rate in buried and surface remains. J Forensic Sci. 2010;55:889–892. 20412365 10.1111/j.1556-4029.2010.01402.x

[27] Mégnin P La faune des cadavres. application de l'Entomologie a la Mèdicine Legale, Paris. Encyclopèdie Sci Aide-Memoire. 1894.

[28] Payne JA A summer carrion study of the baby pig *Sus**scrofa* Linnaeus. Ecology. 1965;46:592–602.

[29] Schoenly K, Goff ML, Early M A BASIC Algorithm for calculating the postmortem interval from arthropod successional data. J Forensic Sci. 1992;37:808–823. DOI:10.1520/JFS11992J 1629673

[30] Le Blanc HN, Logan JG Exploting insect olfaction in forensic entomology. In: Amedt J, Campobasso CP, Goff ML, Grassberger M, editors. Current concepts in forensic entomology. Dordrecht: Springer; 2009 p. 205–221.

[31] Forbes SL, Carter DO Processes and mechanisms of death and decomposition of vertebrate carrion. In: Benbow ME, Tomberlin JK, Tarone AM, editors. Carrion ecology, evolution, and their applications. Boca Raton (FL): CRC Press; 2016 p. 13–30.

[32] Rodriguez W, Bass W Insect activity and its relationship to decay rates of human cadavers in east Tennessee. J Forensic Sci. 1983;28:423–432. DOI:10.1520/JFS11524J

[33] Goff ML, Lord WD Hanging out at the sixteenth hole: problems in estimation of postmortem interval using entomological techniques in cases of death by hanging [abstract]. 46th Annual Meeting of the American Academy of Forensic Sciences; Feb 14–19; San Antonio (TX): AAFS Publisher; 1994 p. 158.

[34] Introna F, De Donno A, Santoro V, et al. The bodies of two missing children in an enclosed underground environment. Forensic Sci Int. 2011;207:40–47. DOI:10.1016/j.forsciint.2010.12.007 21255948

[35] Rodriguez WC Decomposition of buried and submerged bodies. In: Haglund WD, Sorg MA, editors. Forensic taphonomy: the post-mortem fate of human remains. Boston (MA): CRC Press; 1997 p. 459–464.

[36] Johnson AP, Wallman JF, Archer MS Experimental and casework validation of ambient temperature corrections in forensic entomology. J Forensic Sci. 2012;57:215–221. DOI:10.111/j.1556-4029.2011.01900.x 21854385

[37] Dourel GL, Paquerault T, Gaudry E, et al. Using estimate on-site ambient temperature has uncertain benefit when estimating postmortem interval. Psyche. 2010;7 DOI:10.1155/2010/610639

[38] Bugelli V, Campobasso CP Basic research and applied science in forensic entomology. Sci Justice. 2017;57:157–158. 28454622 10.1016/j.scijus.2017.04.006

[39] De Donno A, Campobasso CP, Santoro V, et al. Bodies in sequestered and non-sequestered aquatic environments: a comparative taphonomic study using decompositional scoring system. Sci Justice. 2014;54:439–446. 25498931 10.1016/j.scijus.2014.10.003

[40] Galloway A, Birkby WH, Jones AM, et al. Decay rates of human remains in an arid environment. J Forensic Sci. 1989;34:607–616. 2738563

[41] Galloway A The process of decomposition: a model from the Arizona-Sonoran Desert. In: Haglund WD, Sorg MA, editors. Forensic taphonomy: the post-mortem fate of human remains. Boston (MA): CRC Press; 1997. p. 139–150.

[42] Campobasso CP, Falamingo R, Grattagliano I, et al. The mummified corpse in a domestic setting. Am J Forensic Med Pathol. 2009;30:307–310. DOI:10.1097/PAF.0b013e318187df4b 19696596

[43] Campobasso CP, Marchetti D, Introna F, et al. Postmortem artifacts made by ants and the effect of ant activity on decompositional rates. Am J Forensic Med Pathol. 2009;30:84–87. 19237864 10.1097/PAF.0b013e318187371f

[44] Charabidze D, Colard T, Vincent B, et al. Involvement of larder beetles (Coleoptera: Dermestidae) on human cadavers: a review of 81 forensic cases. Int J Leg Med. 2014;128:1021–1030. 10.1007/s00414-013-0945-1 24292547

[45] Parks CL A study of the human decomposition sequence in central Texas. J Forensic Sci. 2011;56:19–22. 20840291 10.1111/j.1556-4029.2010.01544.x

[46] Heaton V, Lagden A, Moffatt C, et al. Predicting the postmortem submersion interval for human remains recovered from UK waterways. J Forensic Sci. 2010;55:302–307. 20102465 10.1111/j.1556-4029.2009.01291.x

[47] Villet MH, Richards CS, Midgley JM Contemporary precision, bias and accuracy of minimum post-mortem intervals estimated using development of carrion-feeding insects. In: Amendt J, Campobasso CP, Goff ML, Grassberger M, editors. Current concepts in forensic entomology. Dordrecht: Springer; 2010 p. 109–137.

